# Mucoepidermoid carcinoma of the pancreas: A case report and literature review

**DOI:** 10.1097/MD.0000000000036993

**Published:** 2024-01-26

**Authors:** Huan Zhang, Shuyan Wang, Chunnian Wang

**Affiliations:** a Department of Pathology, Ningbo Clinical Pathological Diagnosis Center, Ningbo, China

**Keywords:** adenosquamous carcinoma, CRTC1–MAML2, mucin-producing cells, mucoepidermoid carcinoma, pancreas

## Abstract

**Introduction:**

Primary mucoepidermoid carcinoma (MEC) is a common malignant neoplasm of the salivary glands, but is very rare in the pancreas. To date, only 10 cases have been reported in the literature. Because MEC of the pancreas is very rare, there is little information about its diagnosis, treatment, and metastasis. Herein, we present the eleventh case and review the relevant literature.

**Patient concerns:**

A 65-year-old woman presented with a mass in the body of the pancreas and multiple masses in the liver on abdominal magnetic resonance imaging. The patient initially underwent EUS-guided fine-needle aspiration and was diagnosed with adenocarcinoma. After adjuvant chemotherapy, resection of the pancreatic body and tail was performed, and the tissues were pathologically, histologically, and immunochemically examined. Specific strains and gene rearrangements were analyzed.

**Diagnosis:**

Mucoepidermoid pancreatic cancer.

**Intervention:**

After a 4-month course of adjuvant chemotherapy, laparoscopic surgery was performed.

**Outcomes:**

The patient is alive until the submission of this paper.

**Conclusion:**

We presented a case of mucoepidermoid pancreatic cancer in a 65-year-old woman. Pathological examination revealed that the tumor parenchyma consisted of 3 cell types. There are mainly epidermoid cells, intermediate cells between the basal and epidermoid cells, and mucus-producing cells in varying proportions. Immunohistochemical staining showed that there were different types of cells with unique morphological characteristics. In summary, primary MECs of the pancreas are rare and have poor prognosis. Few studies have been conducted on the diagnosis, treatment, and metastasis of MECs; therefore, further studies are needed to detect them.

## 1. Introduction

Mucoepidermoid carcinoma (MEC) is a relatively common malignant tumor that occurs mainly in the salivary glands. It has also been reported in the digestive tract, respiratory tract, and breast.^[1,2]^ MECs were first reported as an independent tumor entity by Stewart et al^[3]^ The first case of MECs in the pancreas was reported by Franz in 1959.^[4]^ To date, only 10 cases of pancreatic MECs have been reported. Herein, we present the 11th case of mucoepidermoid pancreatic cancer in a 65-year-old woman and review relevant literature.

The characteristics of tumor cells were also studied using immunohistochemical staining, special staining, and fluorescence in situ hybridization (FISH).

## 2. Case report

A 65-year-old woman was admitted to the local hospital because of a cough and underwent a computed tomography (CT) scan that showed multiple space-occupying lesions in the liver. The patient agreed to be hospitalized for further examination and treatment. At the time of admission, she had no obvious discomfort or jaundice, and only slight pain in the abdomen. No significant abdominal mass was found during physical examination. Serum carbohydrate antigen 19-9 (CA19-9) levels increased to 258.5 IU/mL (the normal range is <37 U/mL). However, the serum levels of alpha-fetoprotein, carcinoembryonic antigen, carbohydrate antigen 125, carbohydrate antigen 15-3, and ferritin were normal. Abdominal magnetic resonance imaging revealed a solid mass in the body of the pancreas, approximately 2.6 cm in diameter, and multiple masses in the liver, suggesting pancreatic cancer with liver metastases. The patient initially underwent EUS-guided fine-needle aspiration and was diagnosed with adenocarcinoma. Preoperatively, a 4-month course of adjuvant chemotherapy with paclitaxel, gemcitabine, and tilelizumab was administered. Laparoscopic surgery was performed, and the tumor was detected in the body of the pancreas with metastasis to the liver.

Postoperatively, the patient underwent regular reexamination with a CT scan, which detected a new metastasis to the peritoneum. The patient is alive until the submission of this paper.

## 3. Pathologic examination

**Figure 1.**
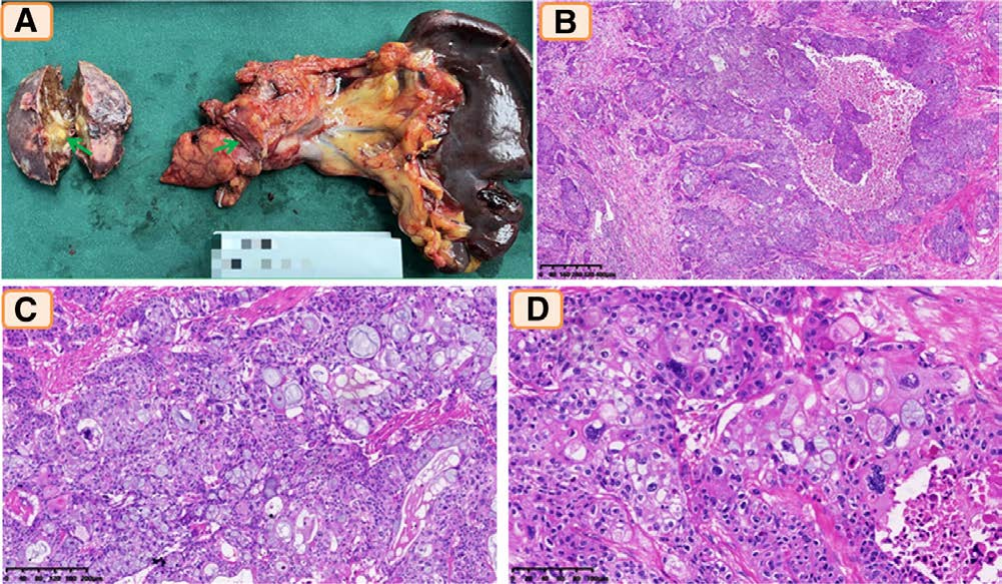
Gross examination: (A) the tumor was in the body of the pancreas, and the cut surface was gray-white, solid and homogeneous; Histological findings: (B) large irregular nests with necrosis surrounded by a dense fibrotic stroma were identified (hematoxylin and eosin [H&E] stain, ×40), (C) there were a number of mucinous cells (H&E stain, ×100), (D) several tumor giant cells could be seen (H&E stain, ×100).

The body and tail of the pancreas, a part of the liver, and the spleen were resected and sent to the pathology department. On gross examination, the tumor was 3.0 cm × 2.5 cm in size in the body of the pancreas, and the cut surface was gray-white, solid, and homogeneous, with a hard elastic consistency (Fig. 1A). In addition, 2 masses in the liver were discovered with a grayish-yellow solid cut surface and hard elastic consistency. The size of the 2 metastases was 2.2 cm and 0.8 cm. Microscopically, the tumor cells in the pancreatic mass were arranged in irregular nests or solid sheets surrounded by dense fibrotic stroma (Fig. 1B). The tumor cells were arranged in glandular tubes. The tumor consisted of 3 types of cells: the majority were epidermoid cells and cells intermediate between basal cells and epidermoid cells, mucin-producing cells were mixed in various proportions (Fig. 1C), and several tumor giant cells were scattered among them (Fig. 1D). These epidermoid cells did not have clear intercellular bridges, and keratohyaline or keratin pearls were not found. Mitoses were observed most frequently, with up to 9 per 10 high-power fields. Meanwhile, we found that the tumor was insensitive to chemotherapeutic drugs and invaded fibrous adipose tissue around the pancreas. However, the metastatic lesion in the liver was sensitive to chemotherapeutic drugs, extensive fibrosis persisted, and only a small number of tumor cells remained.

## 4. Immunohistochemical findings

**Figure 2.**
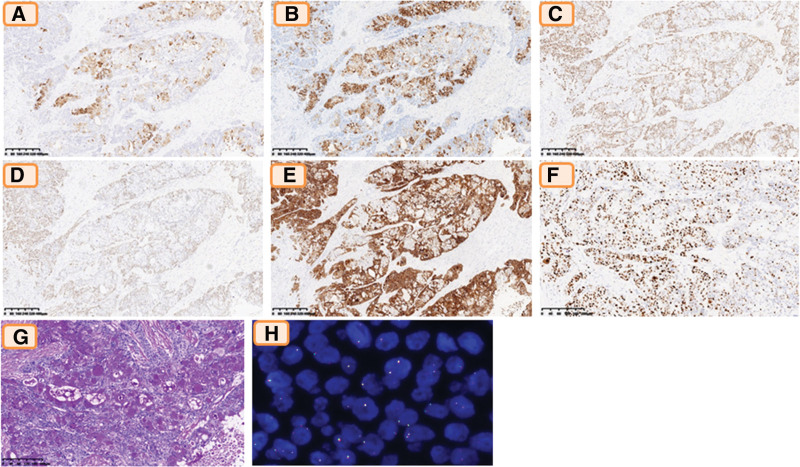
Immunohistochemical results: the mucous cells were focally positive for CK7 (A) and Muc5AC (B), in addition, the epidermoid cells and intermediate cells were positive for p40 (C), p63 (D) and CK5/6 (E); (F) the proliferation index by Ki-67 stain was about 25%; (G) special stains, periodic acid–schiff and mucicarmine showed that there were a lot of mucous cells; (H) CRTC1-MAML2 fusion transcript was not detected by fluorescence in situ hybridization (FISH).

Immunohistochemical staining for CK7(UMAB161, ZSGB), Muc5AC (MRQ-19, ZSGB), P40 (ZR-8), P63 (UMAB4, ZSGB), and CK5/6 (OTI1F8, ZSGB) was *conducted*. The epidermoid cells and intermediate cells were positive for p40, p63, and CK5/6, and the mucous cells were focally positive for CK7 and Muc5AC (Fig. 2A–E). The proliferation index determined by Ki-67 immuno*stain* was approximately 25% (Fig. 2F). Special stains, periodic acid–Schiff, and mucicarmine showed that there were a lot of mucous cells (Fig. 2G). Furthermore, gene rearrangement and split signals were not detected by FISH using an isolated probe of the CTRC-MAML2 gene type (Fig. 2H). MECs are classified as low-, intermediate-, or high-grade according to their histologic features in the salivary gland.^[5]^ Based on diagnostic standards, the final pathological diagnosis was high-grade mucoepidermoid carcinoma.

## 5. Discussion

Primary mucoepidermoid pancreatic cancers are rare entity.^[6]^ MEC has long been considered a subtype of adenosquamous carcinoma, which is histologically composed of both adenocarcinoma and squamous carcinoma.^[7,8]^ However, studies have shown that MECs and adenosquamous carcinomas have different histological characteristics.^[3]^ Histologically, both ductal adenocarcinoma and squamous carcinoma usually account for at least 30% of adenosquamous carcinoma.^[9]^ However, MECs consist of 3 types of cells in varying proportions: mucous cells, epidermoid cells, and cells between basal and epidermoid cells. MECs are thought to originate from intermediate cells.^[10]^ These 3 cell types are always closely mixed and may be accompanied by cystic configurations. Unlike the squamous component found in squamous or adenosquamous carcinomas, epidermoid cells of MECs usually lack keratosis and visible intercellular bridges.^[11]^ In the present case, the tumors were arranged in irregular nests or solid sheets with a fibrotic interstitium. The nucleoli of the epidermoid cells were obvious. Intermediate cells and vacuolar mucous cells accounted for approximately 30%, and a large number of giant tumor cells were observed. Therefore, this case was diagnosed as MEC, but not adenosquamous carcinoma.

According to their morphological and cytological characteristics, MECs in salivary glands are divided into 3 levels: low, medium, and high differentiation.^[5]^ MEC is currently classified using the Armed Forces Institute of Pathology (AFIP) histological classification, which is based on histopathological features including a composite score of cystic components, nerve invasion, necrosis, mitotic activity, and cytological pleomorphism.^[12]^ The AFIP quantitative grading system was based on the sum of the scores for the 5 histopathological features. A score of 0 to 4 is low grade, 5 to 6 is medium grade, and 7 to 14 is high grade (Table 1).^[13]^ High-grade MECs are associated with a higher risk of recurrence, metastasis, and tumor-related death, whereas low-grade MECs usually have a good prognosis and few metastases.^[12]^ According to the AFIP scoring system, our case scored 12 points; therefore, it was classified as high-grade (Table 1).

**Table 1:** The point score and grade of present case according to Armed Forces Institute of Pathology (AFIP) grading system for mucoepidermoid carcinoma.

AFIP grading system	Current case
Histologic parameter (point value)	
Intracystic component ˂ 20% (2)	No
Neural invasion present (2)	Yes
Necrosis present (3)	Yes
Mitosis, 4 or more per 10 high-power fields (3)	Yes
Anaplasia present (4)	Yes
Total point score	12
Tumor grade (point score)	
Low (0–4)	
Intermediate (5–6)	
High (7–14)	Yes

Up to now, only 10 cases of primary MECs of the pancreas have been *described* in the literature (Table 2).^[6,9,14–22]^ The patients included 7 males and 3 females, with ages ranging from 48 to 75 years, with a mean age of 60.7. Three tumors were detected in the head of the pancreas, 4 in the body, and 2 in the tail. At the time of initial diagnosis, the tumor was 3.5 cm and 10 cm in size. All advanced cases showed invasive growth. Furthermore, 5 of the 10 cases displayed metastatic lesions in the liver, which was observed in our case. Cutaneous metastases occur in rare cases.^[9]^ The prognosis was extremely poor in all the cases. Treatments including surgery, radiation, and chemotherapy have proven to be less effective. Based on previously reported cases, if the tumor can be removed intact, surgery seems to be the only way to offer hope for a cure.^[20]^ In the current patient, adjuvant chemotherapy with paclitaxel, gemcitabine, and tilelizumab was administered; however, the tumor was not sensitive to chemotherapy.

**Table 2:** 10 cases of mucoepidermoid carcinoma of the pancreas reported during years 1987 to 2023.

Case	Age (year)/sex	Location	Diameter (cm)	Progression	Operation	Prognosis
Naoki Imazu et al (2021)	75/M	Not mentioned	8.5	Invasion to liver, stomach, celiac artery, portal vein and superior mesenteric vein	Not available	2.8 months, dead
Prasamsa Pandey et al(2016)	50/M	head	8.8	Invasion to cutaneous	Total pancreatectomy	36 months, dead
Li JT et al (2012)	63/F	Body and Tail	4.5	Local pancreatic tumor	Pancreatic body and tail resection	12 months, dead
Li XD et al (2002)	65/M	Body	10.0	Local pancreatic tumor	Not available	Not available
Onoda N et al (1995)	64/M	Tail	8.0	Invasion to spleen, left kidney and adrenal, colon, multiple liver metastases, peritoneal dissemination	Pancreatic tail resection	11 months, alive
Kimura et al (1993)	57/M	Body	8.0	Invasion of stomach, colon, multiple liver and lung metastasis	Intraoperative radiation therapy	2 months, dead
Hayashi et al (1992)	58/M	Head	3.5	Lymph node metastasis	Pancreatoduodenectomy	6 months, alive
Kishimoto et al (1991)	48/F	Body	3.5	Solitary liver metastasis	Total pancreatectomy	11 months, dead
Ohtsuki et al (1987)	58/M	Tail	10.0	Multiple liver metastases	(−)	2 months, dead
Ohshio et al (1987)	69/F	Head	T2	Invasion of mesocolon, superior mesenteric vein	Pancreatoduodenectomy	3 months, dead

The present study confirmed the initial results reported by Behboudi et al^[23]^ that CRTC1-MAML2 gene fusion exists in MECs, especially in low-grade MECs, which have a good clinical prognosis.^[24–27]^ Furthermore, low- and high-grade salivary glands have different genomic changes and CRTC1-MAML2 fusion statuses. Low-grade tumors generally do not have genomic imbalances and are positive for CRTC1-MAML2 gene fusion, whereas high-grade tumors mainly show numerous genomic imbalances and negative gene fusion.^[12]^ In our patient, the final pathological diagnosis was high-grade MEC, and the CRTC1-MAML2 fusion transcript could not be identified using FISH. Of the 10 previously reported cases, only 1 patient underwent CRTC1-MAML2 fusion examination, which was also negative.^[14]^

In summary, primary MECs of the pancreas are rare and have poor prognosis. There are few studies on the diagnosis, treatment, and metastasis of MECs. Further studies are needed to confirm this hypothesis.

## Author contributions

**Data curation:** Huan Zhang, Shuyan Wang.

**Formal analysis:** Huan Zhang, Chunnian Wang.

**Funding acquisition:** Chunnian Wang.

**Investigation:** Shuyan Wang.

**Writing – original draft:** Huan Zhang.

**Writing – review & editing:** Huan Zhang, Chunnian Wang.
